# Integrated methodology for correlating dynamic parameters with wheel wear progression in a scaled railway system

**DOI:** 10.1016/j.mex.2026.103793

**Published:** 2026-01-08

**Authors:** Tania Elizabeth Sandoval-Valencia, Gerardo Hurtado-Hurtado, Luis Morales-Velázquez, Dante Ruiz-Robles, Juan Carlos Jáuregui-Correa

**Affiliations:** a Faculty of Engineering, Autonomous University of Queretaro, Santiago de Queretaro 76010, Mexico; b Escuela Nacional de Estudios Superiores Unidad Juriquilla, Universidad Nacional Autonoma de México, Querétaro 76230, Mexico

**Keywords:** Railway dynamics, Wheel-rail interface, Wear monitoring, Multisensor data acquisition, Scaled testing, Factorial design

## Abstract

Railway wheel wear poses major safety and maintenance challenges, yet accurate predictive models are limited by a lack of synchronized dynamic and wear data from scaled systems. This article presents an integrated methodology to generate a correlative dataset of dynamic parameters and wear progression on the wheels of a 1:20 scale railway system. The experimental approach combines synchronized multisensor data acquisition with sequential microscopic imaging under controlled operating conditions, specifically during braking maneuvers at track transitions. The resulting publicly available dataset enables direct analysis of how operational factors influence physical degradation.

Integration of synchronized sensor data with sequential microscopic imaging to correlate dynamics and wear progression.

Controlled factorial experimental design varying speed and braking zones to ensure reproducible testing conditions.

Publicly available dataset supporting model calibration, predictive algorithm development, and defect quantification for railway maintenance applications.

## Graphical abstract

**Figure:**
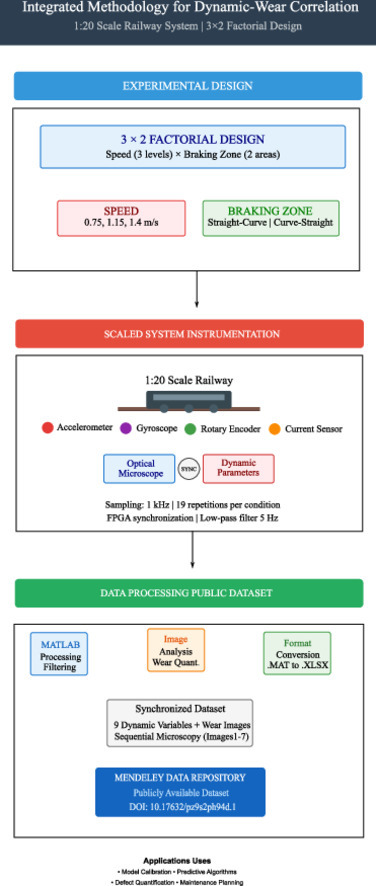

Specifications table

**Table 1:** Specifications.

**Subject area**	Materials Science
**More specific subject area**	*Wear on railway wheels*
**Name of your method**	*Integrated 3 × 2 full factorial experimental method correlating dynamic parameters with wheel wear progression in scaled railway systems*
**Name and reference of original method**	Full factorial experimental design method (customized as 3 × 2 design) based on [1,2].
**Resource availability**	*Equipment and Hardware:* • *TCRT5000 Optical Sensor - For train speed measurement.* • *DCS-24V Rotary Encoder - For angular wheel speed measurement.* • *LSM6DS3 MEMS Accelerometer/Gyroscope - For 3-axis acceleration (±4g) and angular velocity (±143°/s).* • *ACS712 Hall Effect Sensor - For motor current measurement (A).* • *DUA-III Control Card with FPGA (Field-Programmable Gate Array) - For signal processing at 1 kHz.* • *CARYWON® Digital Optical Microscope - For wear image acquisition (1000X magnification).* • *1:20 Scale Railway System - Laboratory-scale test track with straight and curved sections.* *Software and data processing:* • *MATLAB® - For data filtering and processing (low-pass filter, 5 Hz).* • *MAT to .XLSX Conversion - Data format transformation for accessibility.* • *Digital Image Processing Tools - For wear analysis and defect quantification.* *Data repository:* • *Repository: Mendeley Data.* • *Dataset DOI: 10.17632/pz9s2ph94d.1* • *Direct URL:* https://data.mendeley.com/datasets/pz9s2ph94d/1 • *Content: All experimental data (.XLSX files) and wear images (high-resolution chronological series).* *Experimental materials:* • *Zebra Tape - Black-white variation tape for optical speed sensing.* • *Test Railway Wheels - 46 mm diameter wheels (with and without pulley).* • *Power Supply - DCS-24V power for rotary encoder.*

## Background

Wheel wear on railway systems is a major challenge for safety, maintenance, and economic viability [3]. This issue worsens during braking and on curves because friction and wheel-rail slippage increase. Rolling contact wear and fatigue analyses, based on train dynamics, offer an alternative approach [4].

The wheel-rail interface directly affects safety, maintenance costs, and operational efficiency. Analyzing wear mechanisms now means separately studying vehicle dynamics and material deterioration. A complete assessment requires synchronized data that records dynamic behavior and wear progression under controlled conditions.

Full-scale experiments are costly and involve variables that are hard to control, complicating data capture during braking. Scale models offer an efficient alternative.

This methodology was developed to provide a standardized experimental procedure. It enables coordinated acquisition of dynamic parameters and wear evolution in railway wheels. The method focuses on braking during critical track transitions: straight-to-curve and curve-to-straight. Mechanical stresses and wear rates rise in these transitions.

The research meets the need for integrated and public protocols. These unite vehicle dynamics (such as accelerations, velocities, and forces) with experimental tribology (such as sequential microscopy of surfaces). Using a 1:20 scale railway system creates a controlled, reproducible environment. Synchronized multi-sensory data can be captured during induced slip events.

Researchers and engineers can use this methodology to correlate operating conditions with wheel degradation patterns. It also helps calibrate multi-body simulations and develop predictive maintenance models. The approach supports the validation of digital image correlation and machine learning algorithms for defect detection. It provides a structured framework to collect dynamic and visual datasets aligned under repeatable test conditions.

## Method details

The experiment had three phases: configuring sensors and designing tests, conducting controlled tests with synchronized data collection, and organizing data. This process creates a reproducible link between dynamic parameters and wear evolution.

## Railway system and scaling considerations

The 1:20 geometric scale railway system was developed as a methodological testbed to investigate fundamental tribological interactions during braking, rather than as a physical replica for full-scale dynamic prediction (e.g., using Froude or Reynolds numbers). Consequently, while geometric scaling was strictly maintained, the primary objective was not to achieve complete dynamic similarity, but rather to establish a robust framework for correlating dynamic signals with surface wear under repeatable laboratory conditions.

To ensure observable wear progression within reasonable experimental timeframes, a combination of 7075-T6 aluminum wheels and steel rails was selected; this combination intentionally accelerates surface degradation while maintaining a representative slip-to-roll ratio. Furthermore, the geometry uses 46 mm-diameter wheels and 5 mm cylindrical rails to concentrate contact stresses, ensuring a stable Region of Interest (ROI) for sequential imaging. Although the force magnitudes and material responses may differ from those in real systems, the synchronized kinematic interactions and signal patterns provide a high-fidelity dataset for training predictive maintenance algorithms, where data synchronization is more critical than exact physical-scale replication.

Experimental design is essential for obtaining reliable and reproducible results in scientific research. A well-structured experimental design enables the identification and control of variables, minimizes errors, and ensures the validity of the results. The key elements of experimental design are shown in Table 2.

**Table 2:** Key elements of design of experiments [[5], [6], [7]].

Element	Description
Response variables	These are the results measured to evaluate the effects of the studied factors.
Study variables (factors)	These are the parameters that are deliberately manipulated to observe their impact.
Variable levels	These are the values or categories assigned to each factor.
Noise variables	Uncontrolled factors that may influence the response must be minimized or controlled.

Therefore, the following was established:

  • Response variables: Microscopic images that allow the evolution of wear to be evaluated.
  • Study variables: Braking area (curve-straight and straight-curve) and train speed.
  • Noise variables: Humidity and temperature in the laboratory.
  • Variable levels: Three speed levels and two braking areas were defined for the factorial design.

A 3 × 2 full factorial experimental design was chosen (Table 3). In this design, Factor A is the speed of the rail vehicle, with three levels: low (-1, 0.75 m/s), medium (0, 1.15 m/s), and high (1, 1.4 m/s). Factor B is the test's starting and braking track segment, where -1 means the test starts on a straight section and brakes in the first curve, and 1 means the test starts on a curve and brakes on a straight section.

**Table 3:** Experimental matrix.

Run	A	B
Exp1Rec_Cur	0	-1
Exp2Rec_Cur	-1	-1
Exp3Rec_Cur	1	-1
Exp4Cur_Rec	0	1
Exp5Cur_Rec	-1	1
Exp6Cur_Rec	1	1

The experimental matrix (Table 3) shows that the first pass was performed at a speed of 1.15 m/s, the second at a speed of 0.75 m/s, and finally at a speed of 1.4 m/s, all starting on the straight section of the track and braking where the first curve begins (Fig. 3a). The second experimental stage shows that the first pass was performed at a speed of 1.15 m/s, the second at a speed of 0.75 m/s, and finally at a speed of 1.4 m/s, all starting on the curved part of the track and braking on the straight part (Fig. 3b). In each of the experiments, 19 tests were performed. This approach provides valuable information on how different speeds affect the level of wear due to skidding along various braking zones.

The selection of the three speed levels (0.75 m/s, 1.15 m/s, and 1.4 m/s) was determined by the scale platform's operating environment and the acquisition system's signal-to-noise ratio requirements. The upper limit of 1.4 m/s represents the maximum stable speed to ensure safe passage through the 1.94 m-radius curves of the track, preventing derailment due to centrifugal forces. Conversely, the lower limit of 0.75 m/s was set as the minimum threshold for generating dynamic transients that are significantly above the sensor's baseline noise. This range effectively captures the transition between different wear regimes, providing a representative dataset for studying speed-dependent degradation patterns under controlled laboratory conditions.

**Fig. 1:**
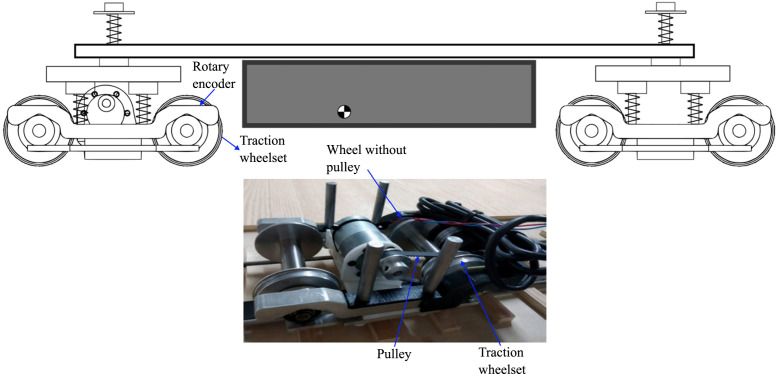
Test area: a) Start in straight part - end in curved part, b) Start on a curved section - end on a straight section.

Furthermore, the selection of three levels distributed across this operating range (low, medium, and high) was made to allow a full factorial design that captures not only the extreme effects but also the trend (linear or non-linear) of wear progression across the system's operating envelope (Fig. 1).

For each experimental condition, 19 consecutive repetitions were performed. This number was determined through a prior convergence analysis, which showed that after 15 trials, the variance of the signal's statistical descriptors (RMS and Mean Energy) had a relative standard deviation of less than 4.2 %, ensuring methodological stability despite subtle ambient noise.

It is important to note that, although each repetition captures a transient dynamic braking response, the sequential execution of the 19 trials primarily aims to measure the accumulation of fatigue and friction damage. Therefore, the results presented should not be interpreted as isolated events, but rather as a consolidated wear state (block of cycles), where the mean signal value represents the dynamic signature of that specific level of deterioration.

### Data acquisition

**Fig. 2:**
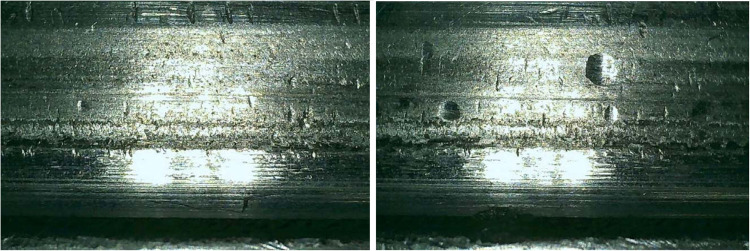
Railway instrumentation.

Raw data is acquired using sensors installed on the test train (accelerometer, gyroscope, current sensor, optical encoder, and rotational encoder), each specialized in monitoring a specific variable (Fig. 2).

  • Accelerometer: Located in the central part of the test vehicle, it is used to measure accelerations in the lateral, vertical, and longitudinal directions of the scale train. The data obtained is in units of g's (one g is equivalent to 9.81 m/s2). The acceleration data is also used to analyze vehicle vibrations.
  • Gyroscope: Used to measure the angular velocities of the train in relation to the three principal axes. This sensor is located on the same chip as the accelerometer. The data obtained is in units of °/s. Angular velocity data is also used to analyze vehicle vibrations, detect curves, and measure roll, pitch, and yaw movements.
  • Current sensor (Hall effect): Used to measure the current flowing through the circuit that powers the motor. The data obtained is in amperes (A). Data from this sensor is also used to measure tangential forces on the drive wheels and torque.
  • Infrared optical sensor: This sensor generates pulses when it encounters black-white variations. It is used in conjunction with a tape featuring black-and-white variations (colloquially known as zebra tape) installed on the test track to measure the vehicle's position and speed at any given moment. To measure speed, preprocessing is required that considers the width of the black-white variations on the tape and the time, in m/s, between each pulse.
  • Rotary encoder: This is used to measure the angular speed of the train's drive wheels. The rotary encoder generates a series of pulses that correlate with the wheel's rotation speed. The train's microcontroller monitors these pulses and stores them as pulses per second (p/s), so external preprocessing is required to convert this information into the wheel's angular speed.
  • Portable optical microscope: Allows image acquisition for monitoring wear evolution (pores, cracks, undercuts).

The FPGA-based triggering system ensured simultaneous activation of all sensors at a 1 kHz sampling rate. Temporal synchronization was verified during post-processing. As the train began to move, data acquisition commenced. Braking, by contrast, was triggered automatically at predetermined transition zones.

All data obtained from the sensors is downloaded from the SD memory into a data file with a .MAT extension. This data is then preprocessed in MATLAB® with a low-pass filter, and these filtered files are converted to an .XLSX extension so that they are accessible and structured. Finally, they are uploaded to the Mendeley repository. Table 4 shows the main functions and typical applications of the built-in sensors.

**Table 4:** Types and functions of vehicle sensors.

Sensor type	Main function(s)	Typical applications	Citations
Accelerometer and gyroscope	Measure acceleration (lateral, vertical, longitudinal) and angular velocity.	Vibration analysis, motion tracking, vehicle classification, localization, roll/pitch/yaw detection.	[8,9]
Current sensor (Hall Effect)	Measure electrical current, infer torque and tangential forces.	Motor monitoring, drive analysis.	[10]
Optical/infrared sensor	Detect position and speed via track/tape or visual cues.	Speed measurement, object detection.	[10,11]
Rotary encoder	Measure wheel rotation and angular speed.	Wheel speed, displacement or position of a vehicle.	[9,12]
Camera	Capture images for object detection, lane keeping, and environment perception.	Obstacle detection, vehicle recognition.	[13]

The methodology generates comprehensive data sets in two formats: dynamic parameters (.XLSX files with nine synchronized variables) and wear evolution images (microscopic sequences). This information enables direct correlation between operating conditions and physical degradation patterns, facilitating various railway engineering applications.

## Quantitative wear measurement

To ensure that wear progression is objectively measurable and reproducible, a digital image processing protocol structured in three consecutive phases has been implemented:

• Definition and preprocessing of the Region of Interest (ROI): To ensure repeatability, a fixed ROI is defined that isolates the tread contact patch, located at coordinates x=30 %, y=28 %, with dimensions of 40 % width and 20 % height relative to the total capture size. Before analysis, a grayscale conversion and Gaussian filtering (5 × 5) are applied to reduce thermal noise from the camera and smooth the image.
• Hybrid defect segmentation: In order to capture the multiscale nature of the damage (from pitting to microcracks), two techniques are executed in parallel:
  ■ Adaptive thresholding: To detect extensive areas of material loss and variations in surface reflectivity.
  ■ Canny edge detection: To segment fine contours and crack boundaries with high precision.5 Both binary masks are merged using a logical OR operation.6 Subsequently, morphological opening and closing operations are applied with a 3 × 3 pixel structuring element to remove noise artifacts and consolidate the segmented damage regions.
• Wear metric calculation: The level of degradation is quantified as the ratio of damaged pixels to the total analysis area, expressed as the percentage of worn area ($A_{w}$).
  $$A_{w}\left(\%\right)=\left(\frac{Segmented\;pixels}{Total\;ROI\;pixels}\right)x100$$

This protocol provides a scalar metric that exhibits a monotonically increasing trend with increasing operating cycles, allowing a physical correlation with vibration energy characteristics.

## Demonstration of correlative analysis

**Fig. 3:**
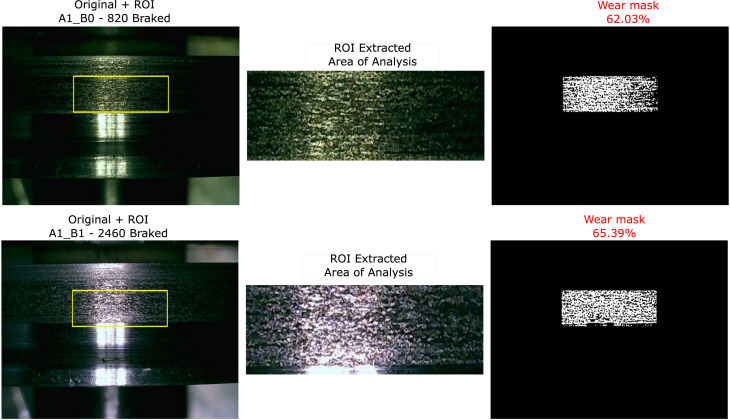
Example of the wear quantification algorithm applied to two different wheel states (820 and 2460 braking cycles).

In a correlative analysis, the methodology was applied in a subsequent experimental laboratory study to estimate Remaining Useful Life (RUL). From the chronological wear images in the repository (images 1-7), the percentage of worn area was objectively quantified using a digital processing algorithm that applies adaptive thresholding and Canny edge detection within a fixed region of interest, as illustrated in Fig. 3. Simultaneously, 12 statistical features were extracted from the synchronously acquired longitudinal acceleration signal (AceXTre_SisFebLab). The analysis revealed a significant correlation (>0.79) between the wear metrics and the vibration characteristics, with the mean vibration energy showing the strongest correlation at 0.94. This result confirms that the dynamic parameters captured using the proposed methodology are physically linked to the progression of surface damage, validating its suitability for direct correlative analysis.

Fig. 3 shows: (left) the original image with the Region of Interest (ROI) defined, (center) the extracted analysis area, and (right) the resulting wear mask with the percentage of damaged area calculated automatically.

## Resource availability

All resources necessary to replicate this methodology are publicly available. The dataset in the Mendeley repository (10.17632/pz952ph94d.1) provides complete experimental results with the following organization:

Data from the experiment in which the train started on the straight section of the track and braked at the beginning of the first curve:

  • Exp1Rec_Cur. The train was run at a speed of 0.75 m/s. Nineteen runs were made.

The images acquired with a microscope before starting the test are named in a folder as images1. These are divided into two folders (pulley and without pulley), and the images acquired at the end of the tests are in the images2 folder.

  • Exp2Rec_Cur. The train ran at a speed of 1.15 m/s. Nineteen runs were made.

The images acquired with a microscope before starting the test are those containing image2 (from experiment 1), and those acquired at the end of the tests are images3.

  • Exp3Rec_Cur. The train was running at a speed marked on the cell phone as 1.4 m/s. Nineteen runs were made.

The images acquired with a microscope before starting the test are images3 (from experiment 2), and those acquired at the end of the test are images4.

Data from the experiment in which the train started on the curved part of the track and braked on the straight part:

  • Exp4Cur_Rec. The train was run at a speed set on the cell phone to 0.75 m/s. Nineteen runs were made.

The images acquired with a microscope before starting the test are those containing images4 (from experiment 3) and those acquired at the end of the tests, images5.

  • Exp5Cur_Rec. The train was running at a speed marked on the cell phone as 1.15 m/s. Nineteen runs were made.

The images acquired with a microscope before starting the test are those containing images5 (from experiment 4), and those acquired at the end of the tests are images6.

  • Exp6Cur_Rec. The train was running at a speed marked on the cell phone as 1.4 m/s. Nineteen runs were made.

The images acquired with a microscope before starting the test are those labeled as images6 (from experiment 5), and those acquired at the end of the test are labeled as images7.

Within the same folder, you will find a folder containing the experimental data for each test, which were named consecutively (Table 5).

**Table 5:** File labels.

File code	Description
Exp1Rec_Cur	General folder, Experiment 1: Rec_Cur
Exp2Rec_Cur	General folder, Experiment 2: Rec_Cur
Exp3Rec_Cur	General folder, Experiment 3: Rec_Cur
Exp4Cur_Rec	General folder, Experiment 4: Cur_Rec
Exp5Cur_Rec	General folder, Experiment 5: Cur_Rec
Exp6Cur_Rec	General folder, Experiment 6: Cur_Rec
Exp1RecCur_Din_SisFebLab	Train dynamics, Experiment 1: Straight-Curve
Exp2RecCur_Din_SisFebLab	Train dynamics, Experiment 2: Straight-Curve
Exp3RecCur_Din_SisFebLab	Train dynamics, Experiment 3: Straight-Curve
Exp4CurRec_Din_SisFebLab	Train dynamics, Experiment 4: Curve-Straight
Exp5CurRec_Din_SisFebLab	Train dynamics, Experiment 5: Curve-Straight
Exp6CurRec_Din_SisFebLab	Train dynamics, Experiment 6: Curve-Straight
images1	Images before starting the test, Experiment 1
images2	Images after completing the 19 experimental runs, Experiment 1
images3	Images after completing the 19 experimental runs, Experiment 2
images4	Images after completing the 19 experimental runs, Experiment 3
images5	Images after completing the 19 experimental runs, Experiment 4
images6	Images after completing the 19 experimental runs, Experiment 5
images7	Images after completing the 19 experimental runs, Experiment 6

**Fig. 4:**
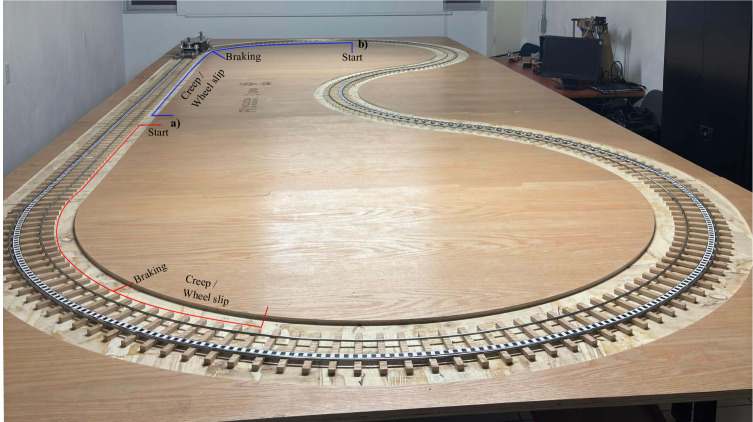
Wheel with and without pulley.

The railway car features a rotary encoder in the drive bogie with a pulley, which is crucial for analyzing wheel dynamics during events such as braking or rapid acceleration. Since both the wheel with pulley (associated with the drive bogie and encoder) and the wheel without pulley are susceptible to significant damage during braking maneuvers, it was decided to acquire images of both. This strategy allows for a comprehensive assessment of wear on the wheels that experience the most significant stresses. Including images of both wheels (with pulley and without pulley, as shown in Fig. 4) provides a more complete view of the evolution of wear on critical components of the railway system at scale.

The labels found at the top of the data obtained from the dynamics of the 1:20 scale railway train (Exp1RecCur_Din_SisFebLab, Exp1RecCur_Din_SisFebLab, and the others) are shown in Table 6.

**Table 6:** Dynamic variables in .XLSX files.

Variable code	Description
VelTre_SisFebLab	Train speed (m/s)
VelRueTra_SisFebLab	Drive wheel speed (m/s)
AceXTre_SisFebLab	Acceleration of the train body in x (g)
AceYTre_SisFebLab	Acceleration of the train body in y (g)
AceZTre_SisFebLab	Acceleration of the train body in z (g)
VelXAngTre_SisFebLab	Angular velocity of the train body in x (°/s)
VelYAngTre_SisFebLab	Angular velocity of the train body in y (°/s)
VelZAngTre_SisFebLab	Angular velocity of the train body in z (°/s)
CorrMot_SisFebLab	Current consumed by the motor (A)
RueDes_SisFebLab	Wear evolution images

**Fig. 5:**
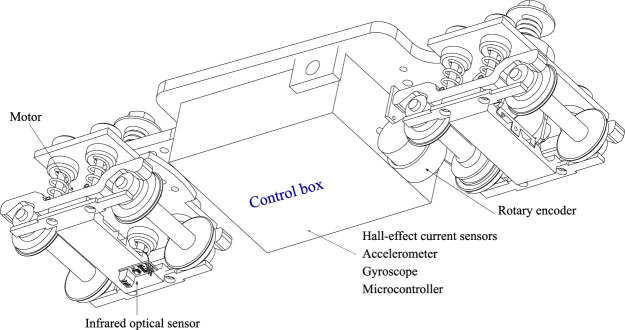
Wear on railway wheels.

Fig. 5 shows examples of observed railway wheel wear. These images illustrate the type of data collected and the nature of the wear. They highlight how wear evolves at the microscopic level. This enables both qualitative and quantitative analysis of the dataset.

Fig. 4 shows two high-resolution digital optical microscopy images of the wheel profile, capturing wear evolution. In the left image, irregularities and rough textures indicate initial wear. The right image shows similar wear, with damage concentrated at a specific point, likely a pore or pit from repeated contact and sliding. Both images highlight surface damage, such as pores, pitting, or cracks, relevant to wear progression studies.

This structured dataset gives researchers direct access to synchronized dynamic-wear data for model calibration, algorithm development, and comparative studies in railway tribology.

## Method validation

Methodological validation was carried out through a process that included multiple consistency checks and reproducibility studies. To ensure precise signal synchronization, the optical encoder pulses were systematically compared to the acceleration peaks recorded during braking.

The data obtained showed remarkable consistency: the nineteen (19) repetitions performed for each condition yielded highly reproducible dynamic patterns.

The study confirmed the validity of the comprehensive approach. How? By establishing a direct correlation between operating conditions and physical deterioration. The progression of wear, documented in the sequence of microscopic images (images 1 to 7), showed clearly systematic patterns directly related to the number of braking cycles and the operating conditions imposed.

The most relevant validation datasets are already available in the repository files (e.g., Exp1RecCur_Din_SisFebLab.xlsx). These resources include the raw and filtered signals, along with the associated wear images. Data integrity during the acquisition and processing phases is verified by reviewing these files.

Finally, the complete dataset structure, detailed in Table 5, Table 6, demonstrates that synchronized (dynamics-wear) correlations can be generated in a rigorously controlled experimental setting. Standardizing file nomenclature and variable labeling ensures that other researchers can easily access and interpret these data for future research.

## Limitations

Variables such as speed and braking zone were controlled. Laboratory conditions minimized noise variables like humidity and temperature. However, subtle, unquantifiable environmental influences may still have affected data consistency across the 19 repetitions for each test. Finally, wear images were acquired after each test, producing a time series of damage. This method did not allow for continuous, real-time monitoring of the wear process during experimental runs.

## Ethics statements

The data resulting from the experiment do not involve human subjects, animal experiments, or information from social media platforms.

## Declaration of generative AI use

In preparing this work, the authors used DeepSeek AI to assist in reviewing the article's format, structure, and suitability for the MethodsX journal. After using this tool, the authors revised and edited the content as needed and take full responsibility for the content of the published article.

## For a published article

None.

## CRediT authorship contribution statement

**Tania Elizabeth Sandoval-Valencia:** Conceptualization, Methodology, Writing – original draft. **Gerardo Hurtado-Hurtado:** Validation, Data curation. **Luis Morales-Velázquez:** Software, Supervision. **Dante Ruiz-Robles:** Supervision, Writing – review & editing. **Juan Carlos Jáuregui-Correa:** Supervision, Writing – review & editing.

## Declaration of competing interest

The authors declare that they have no known competing financial interests or personal relationships that could have appeared to influence the work reported in this paper.

## Data Availability

The data have been shared for free access at https://data.mendeley.com/datasets/pz9s2ph94d/1.
